# Global Unbiased Determination Of Comorbidity (GUDC) In U.S. Older Adults

**DOI:** 10.1093/geroni/igab046.3058

**Published:** 2021-12-17

**Authors:** Beth Hogans, Bernadette Siaton, Raya Kheirbek, Leslie Katzel, John Sorkin

**Affiliations:** 1 VAMHCS, Baltimore, Maryland, United States; 2 University of Maryland School of Medicine, Baltimore, Maryland, United States; 3 University of Maryland School of Medicine, University of Maryland School of Medicine, Maryland, United States; 4 University of Maryland, Baltimore VA Medical Center, Maryland, United States

## Abstract

With age, many adults develop multiple comorbid conditions; and resulting clinical complexity increases markedly so that identifying how specific conditions effect others remains important. Here, our primary objective was rapid unbiased appraisal of pair-wise condition-specific comorbidity; our second objective was identification of common conditions with highest and lowest rates of such comorbidity. In 2016, utilization of ICD-10 codes became mandatory for providers rendering care to Medicare beneficiaries. Universal adoption of ICD-10 coding ensued and concomitantly, all patients had ICD-9 codes replaced with new codes, so that 2017 data represent an opportunity to examine massive amounts of ‘freshly’ coded patient claims data. Evaluating ICD-10 coding data at individual and population levels, we appraised how often two codes were utilized together, i.e. estimated pair-specific comorbidity. Expanding this computationally, we determined the extent to which any given condition was co-coded with all other utilized diagnostic codes, i.e., estimated global, unbiased pair-wise comorbidity. We term this metric the global unbiased dyadic comorbidity (GUDC) value. Based on 40 million claims for a representative sample of 1.5 million older adults across the U.S., GUDC values varied with age and gender but were highly stable across varying comorbid condition prevalence, e.g., common (>1%) vs. less common (1/1000-1/100) prevalence. GUDC values for HIV in older adults were modest, compared to high values for ARDS, we infer substantive progress in HIV management among older adults. We discuss the interpretation and potential applications of GUDC and conclude that access to comorbidity appraisals may advance geriatric care, more study is needed.

